# Helping people to discontinue antipsychotics: if, when and how

**DOI:** 10.1038/s41537-025-00695-x

**Published:** 2025-12-17

**Authors:** Joanna Moncrieff, Mark Abie Horowitz

**Affiliations:** 1 Division of Psychiatry, University College London, London, UK; 2 Research and Development Department, North East London NHS Foundation Trust (NELFT), Essex, UK; 3 Institute of Pharmaceutical Sciences, King’s College London, London, UK

**Keywords:** Schizophrenia, Psychosis

## Abstract

The default position in the treatment of psychotic conditions is that lifelong treatment is required after multiple episodes, with some portion of patients able to stop after a single episode. However, there are reasons to question this consensus, including that not everyone who stops antipsychotics relapses, the protective effects of antipsychotics may have been exaggerated due to the possibility that withdrawal itself is associated with adverse effects, including relapse, there is little evidence that antipsychotics target a pathological mechanism and their adverse effects may outweigh their benefits for some users. Recent pragmatic trials that employ a gradual reduction strategy give mixed results. They highlight that relapse is still a potential risk, but there may be gains in terms of social functioning in the long-term. There are no consistent factors that enable identification of people who might be able to discontinue antipsychotics more successfully, suggesting this option should be more widely offered. A gradual, hyperbolic approach to tapering has been proposed to reduce the adverse effects of withdrawal and relapse on stopping—approximated, for example, by reductions of 5-10% of the most recent dose every month. Patients should be supported to make decisions about long-term antipsychotic treatment based on weighing up the pros and cons of antipsychotic effects, in the light of the evidence base and the individual’s priorities. Given that increasing duration of treatment is likely to increase the risk of withdrawal on stopping, long-term antipsychotic treatment should be minimized where possible.

## Helping people to discontinue antipsychotics: if, when and how

Increasing emphasis on patient choice and quality of life and questions about the evidence base for long-term antipsychotic treatment have provoked interest in possibilities for helping patients to reduce or discontinue such treatment. We look at the reasons for doing this, the timing and the best methods for supporting antipsychotic reduction and discontinuation.

## If: the rationale for discontinuing antipsychotic treatment

Most people diagnosed with schizophrenia or psychotic disorders are prescribed long-term, often lifelong, antipsychotic treatment. Historically, this practice was established almost as soon as antipsychotics were introduced and for years it was unquestioned^1^. Recently, however, there has been more focus on the adverse effects of antipsychotics and patients’ perspectives, which has led to debate about whether long-term treatment is always the best option^2^. Despite this, current guidelines continue to recommend it either explicitly or implicitly for most people with psychotic conditions^3–5^.

Since there is no question that antipsychotics cause a high burden of debilitating and harmful adverse effects, it is important to clarify the reasons why long-term treatment might be beneficial, and to scrutinise the evidence for this supposition. This will, in turn, inform whether people who are already taking antipsychotics should be recommended or supported to try and stop them.

The evidence that justifies long-term antipsychotic treatment principally consists of randomised discontinuation trials, in which people who are already established on antipsychotics are assigned to continue taking them or to have them stopped – with or without placebo substitution^6,7^. Such studies explore whether continuing to take antipsychotics influences the risk of having a ‘relapse’ or deterioration of symptoms.

As Barnes et al. (2020) point out: “A relapse of psychotic symptoms can be a distressing, disruptive and stigmatising experience, jeopardising personal relationships as well as education or employment status and carrying the risk of harm by patients to themselves or others”^5^(P 13–14).

However, although many people experience relapse in this way^8^, it is increasingly recognised that people value other aspects of recovery, such as functional capacity and the fulfilment of personally meaningful roles as well as symptom control and relapse prevention^9,10^.

Additionally, there has been greater interest in the notion of gradual hyperbolic tapering—whereby doses are decreased to produce a linear reduction of effect on target receptors, requiring incrementally smaller dose reductions, down to tiny final doses—with the possibility that this might reduce the risk of relapse on dose reductions, supported by some emerging evidence^11,12^.

## Does long-term antipsychotic treatment prevent relapse?

Randomised trials consistently show that people who discontinue long-term antipsychotic medication are more likely to have a relapse or deterioration of symptoms than those who continue to take it. Although relapse is not inevitable, meta-analyses suggest that people who discontinue antipsychotics are at least twice as likely to relapse^6,7,13,14^. However, this does not necessarily demonstrate that starting on long-term treatment is better than not doing so. Indeed, studies suggest that some people can do well without or with minimal antipsychotic medication^15–17^. The only two existing modern RCTs comparing placebo to antipsychotics treatment in first episode psychosis (combined with psychosocial support in both groups) showed no difference in functioning or symptomatology at 24-month follow-up (notably, patients at high risk of suicide or violence were excluded from these studies, and one was powered as a feasibility study)^15,17^.

Moreover, traditional randomised trials of maintenance treatment demonstrate that a varying proportion of people (up to 40%)^6^ do not relapse following antipsychotic discontinuation, even with abrupt or relatively abrupt discontinuation. Intermittent treatment studies, which employ a discontinuation design followed by intervention for early signs of relapse, also show that although relapse rates are higher than for maintenance treatment, many people do well with this regime^18^. 64.7% of people randomised to intermittent treatment remained relapse free in trials lasting longer than 6 months in the last meta-analysis to provide such figures^18^.

Moreover, rates of relapse may be exaggerated by the adverse effects of the withdrawal process itself. We know that stopping a drug that has been taken on a long-term basis is not the same as never having taken the drug. Long-term use leads to neuroadaptations including, in the case of antipsychotics, dopamine receptor supersensitivity and other modifications that reflect the receptor targets of individual agents^12,19,20^. Dopamine supersensitivity has also been linked to tardive dyskinesia and ‘tolerance’ or reduced efficacy of antipsychotics^20^, exemplified by studies finding relapse rates of 30% while on stable doses of medication in clinical populations^21,22^, predicted by the emergence of tardive dyskinesia^21^. There is evidence that increased dopaminergic sensitivity can occur after days or weeks of treatment in animals^23,24^. Indeed in one carefully designed study rodents withdrawn from two weeks of antipsychotic treatment were found to have 63% higher levels of amphetamine-induced locomotion than control animals, demonstrating clear withdrawal effects, alongside evidence of increased number and sensitivity of dopaminergic receptors^25^. Although there are limitations in extrapolation from animal studies to humans this suggests a mechanism by which antipsychotic exposure may lower the threshold for psychotic relapse after discontinuation.

Some small studies also suggest increased D2/D3 dopamine receptor availability in humans exposed to antipsychotics^26^, with one study quantifying this increase as 30% compared to drug naïve subjects^27^. These neuroadaptations have been shown to persist for months and years in animal studies, mirroring the persistence of tardive dyskinesia in patients who have ceased antipsychotics^12^. The recent findings of the HAMLETT study may provide clinical validation of this phenomenon, with patients treated with partial agonists showing half the risk of relapse compared to high affinity D2 antagonists, suggesting a key role for dopaminergic hypersensitivity in determining relapse^28^

Cessation of antipsychotics can lead to withdrawal symptoms, which include agitation, anxiety, aggression and insomnia, which may be mistaken for non-specific signs of relapse^29^ (particularly since relapse is often defined broadly by an increase in such symptoms^30^). Moreover, case reports indicate that withdrawal can be associated with a new onset of psychotic symptoms in people taking antipsychotics with no prior history of psychotic symptoms and people taking other D2 dopamine receptor blockers for nausea or lactation difficulties with no history of mental disorder^12,31^. It has also been suggested that drug withdrawal may precipitate or bring forward an episode of the underlying condition itself^12,31–33^. The strongest evidence for this consists of studies showing that the risk of having an episode of bipolar disorder, particularly mania, is higher following the discontinuation of lithium than it is before lithium is started^34,35^. Lithium may act in a different manner but the principle of the withdrawal of long-term drug treatment precipitating a relapse of a recurrent condition may be more widely generalisable given the tendency of many different types of drugs to induce biological adaptations which can provoke physical and psychological symptoms when the drug is withdrawn^36^.

For antipsychotics, the possibility that the process of withdrawal increases the risk of relapse or apparent relapse above the natural history of the condition is supported by some evidence, including that the elevated risk of relapse following discontinuation attenuates over time (which is consistent with a front-loaded withdrawal effect)^6,37,38^. In one meta-analysis, 50% of relapses occurred in the first three months following switch to placebo, for example^39^. More gradual tapering, which has been shown to reduce withdrawal effects^40^, reduces the risk of relapse in meta-anlyses^41,42^, but not when it is only over a few weeks^6^. Consistent with this, some studies find that stopping depot preparations, which are excreted more gradually^43,44^, are associated with lower risks of relapse compared to stopping oral medication, including a Cochrane meta-analysis^45–47^.

In contrast, a recent study by McCutcheon et al.^48^ involving a re-analysis of existing data for paliperidone found that the risk of relapse was not dependent on the rate of discontinuation (whether discontinued from oral or depot medication) and that relapses did not occur earlier on oral medication compared to depot medication. However, McCutcheon et al. compared relapse rates within trials using relative rates (discontinuation vs maintenance), rather than absolute relapse rates between oral and depot discontinuation arms. This obscures differences between trials of oral and depot formulations, because adherence is typically poor in oral maintenance groups (inflating relapse in that group), while adherence is much higher in depot maintenance. For example, relapse rates in oral maintenance groups are 29% from Leucht’s meta-analysis^47^, while the relapse rate for maintenance paliperidone is 10%^49^. As a result, the relative risk of relapse on stopping oral medication is minimised, and the gap between oral and depot discontinuation is artificially narrowed.

Another comparison of absolute relapse rates across groups, using a data-set that overlapped with McCutcheon’s, found that depot discontinuation resulted in a 5-fold lower hazard ratio for relapse than oral discontinuation (absolute rate of relapse of 42.3% vs 88.7% for patients who were followed for at least five half-lives after medication cessation)^46^. This is in line with other studies, including a Cochrane meta-analysis, which found that depot discontinuation reduced the risk of relapse compared to oral discontinuation (RR 0.30 vs RR 0.43)^47^. Finally, the association between slower discontinuation and lower relapse risk has been shown for other psychiatric drugs in other conditions^50,51^.

On the other hand, evidence that the pattern and trajectory of symptoms following antipsychotic discontinuation is the same as that in people who relapse while continuing drug treatment, argues against a simple withdrawal state^52^, as does the fact that symptom deterioration usually occurs gradually rather than suddenly following antipsychotic discontinuation^38,45^, although some studies find relapse is rapid in onset^53^. Furthermore, emerging evidence on antidepressants suggests withdrawal effects may sometimes have a delayed onset^54^, highlighting how poorly we understand the consequences of long-term drug treatment and withdrawal, which may have relevance to antipsychotic withdrawal as well.

However, this evidence does not refute the possibility that antipsychotic withdrawal may precipitate, or bring forward an episode of the underlying condition, in the same way that lithium appears to in people with bipolar disorder. The mechanism may be to do with the intensified emotional reactivity or insomnia associated with antipsychotic withdrawal as described by those reducing antipsychotics^55^, lowering the threshold for what precipitates a psychotic episode (e.g. recreational drugs, stress). These vulnerabilities are likely to persist as long as neuro-adaptations to the drug persist, which can be months or even years after cessation^12^.

Therefore, randomised trials of antipsychotic discontinuation may partly reflect the adverse effects of withdrawing from the drug rather than the benefits of taking it, exacerbated by the fact that the vast majority of existing trials involve abrupt or relatively abrupt discontinuation of the antipsychotic and relatively short follow-up^12^. They also highlight that a proportion of people do not relapse after discontinuing treatment.

## Naturalistic studies

The possibility that the benefits of antipsychotics in randomised trials partly reflect adverse withdrawal effects is consistent with evidence from several naturalistic cohort studies that suggest that people who take ongoing antipsychotic treatment have worse long-term outcomes than those who do not^56–59^. These results are likely to be explained partially, at least, by the fact that people with a poor prognosis are more likely to remain on treatment (confounding by severity or indication). However, in a US study of 145 patients with a first episode of psychosis, the negative effects of antipsychotics remained significant when various prognostic indicators (e.g. type of onset, presence of a precipitant, premorbid work and social adjustment) were controlled for^60^. In a Danish study of 303 participants, people in symptomatic remission 10 years after a first episode of psychosis showed better social functioning if they were not taking antipsychotics than if they were^59^.

In contrast, studies using population databases suggest that people who take long-term antipsychotics have better outcomes^61,62^. However, these studies have been criticised on a number of grounds, including that the use of person years as the denominator obscured actual disadvantages for people taking antipsychotics^63–65^. Another naturalistic study found that people who discontinued antipsychotic medication were more likely to relapse than those who maintained it, although there were no differences in symptoms and functioning at 10-year follow-up^66^. Although confounding by indication limits the conclusions that can be drawn from all naturalistic studies, they do provide confirmatory evidence that some patients can remain well without ongoing antipsychotic medication.

## Mechanisms of action

How antipsychotics are thought to work also shapes justifications for taking them and for stopping them. Traditionally, they have been believed to work by targeting an underlying pathological process, in what one of us has described as a ‘disease-centred’ model of drug action^67^. Among other theories, this has been suggested to be by correcting an excess of dopamine by blocking dopamine D2 receptors, in line with the dopamine hypothesis of schizophrenia. However, effects of antipsychotics that have relatively lesser effects on the D2 receptor, such as clozapine and quetiapine^68^, raise questions about this hypothesis and there is still a lack of conclusive evidence of an abnormality of dopamine activity in treatment-naïve people diagnosed with psychosis or schizophrenia^69–71^.

The disease-centred model leads to the assumption that drug treatment is beneficial (other things, such as side effects, being equal) because it is thought to normalise abnormal neurochemistry, or other underlying biological abnormalities. An alternative way to conceptualise the effects of antipsychotics—the ‘drug-centred’ model of drug action - has different implications for making decisions about the value and place of drug treatment. This is the idea that rather than targeting an underlying disease process, antipsychotics suppress symptoms by altering normal brain functioning, for example lowering normal dopamine signalling along with other neurochemical changes. These changes produce a drug-induced state characterised by cognitive and emotional restriction, and varying degrees of sedation, that can reduce symptom intensity^72^. According to this model, treatment needs to be judged by the balance of positive and negative effects on the individual. Within this framework, it is understandable that drugs that may be useful to manage symptoms in an acute episode may have net harmful effects in the long-term, when psychotic symptoms are less troubling, if they impair functioning through sedation and cognitive inhibition, for example.

A variant of the disease-centred model of drug action is the idea that antipsychotics can prevent the ‘disease progression,’ which has been proposed to be induced by psychotic episodes or relapses^5^. It is also suggested that people become less responsive to treatment with successive episodes^53,73^.

Evidence for the idea that acute psychotic symptoms or relapse is a ‘biologically toxic’^74^ (p 347) or ‘degenerative process’^75^ (p 736) is limited^76^. Although there is an association between relapse and poor psychosocial outcomes, lack of remission^77,78^ and a lesser response to antipsychotics following relapse^79,80^, these findings are likely to be confounded by the underlying severity of the condition. Although some longitudinal studies suggest people who relapse have worse long-term outcomes^66,81^, the majority find that symptoms revert to baseline levels after relapse^77–80,82^. Although antipsychotics could have been re-started in these studies, they nevertheless suggest that the deterioration is not permanent (as suggested by the biological toxicity hypothesis). Recent data from the RADAR randomised trial of antipsychotic reduction also showed that people who relapsed did not differ from people who did not relapse in their outcomes at two-year follow-up, including social functioning scores, quality of life, symptoms scores and overall rates of employment^83^.

Some research suggests that although most patients return to baseline following relapse, a small proportion show a deteriorating course^82^. This is also likely to be explained by the underlying nature of the condition to some extent, but it may also reflect the consequences of antipsychotic withdrawal, analogous to the protracted withdrawal states that have been reported following benzodiazepine and antidepressant withdrawal^84–87^. These have been suggested to be a consequence of neurological disruption and damage caused by long-term use followed by withdrawal, especially rapid withdrawal, sometimes called “kindling”(reflecting the effect of drug exposure and withdrawal rather than the effect of relapse per se)^86–89^. Overall, however, a recent narrative review concluded that ‘the conventional notion that relapse leads to treatment non-response or worse long-term outcomes is generally not supported by the highest quality studies’^76^.

## Adverse effects and patient priorities

Although adverse effect profiles vary between individual antipsychotic classes and agents, neurological, cardiac and metabolic adverse effects are well-known. In recent years, evidence has accumulated that antipsychotics might cause brain volume reduction^90–92^, which has been found to be associated with impairment of cognitive functions in some studies, but not others^93^. However, a recent study of people with a first episode of psychosis followed up for a maximum of 12 months showed no clear evidence of volume reduction^94^.

In addition to these complications, antipsychotics cause sexual dysfunction and dysphoria and are often intensely disliked by those who take them^72^. People have described antipsychotics as making them feel ‘braindead,’ like a ‘zombie’ or of creating a ‘living hell’. Some people feel medication side effects outweigh any potential benefits^95^, and many people would like to try to stop taking them at some point^95,96^.

## Recent studies of more gradual antipsychotic reduction and discontinuation

The lack of certainty about the intrinsic benefits of antipsychotics, accumulating evidence of adverse effects and increasing attention to patient preferences has led to increased interest in the pros and cons of helping people to come off antipsychotics. A number of randomised studies of antipsychotic discontinuation have therefore been initiated involving more gradual dose reduction than previous clinical trials and with an emphasis on functional outcomes and medium or long-term follow-up (Table 1)^97,98^.

**Table 1:** Recent RCTs of more gradual antipsychotic reduction.

Study paper	Condition	Numbers randomised and duration of follow-up	Reduction/discontinuation of antipsychotics	Results at early follow-up	Long-term follow-up (if conducted and published)
Wunderink 2007, 2013 (MESIFOS study)	First episode psychosis	At 18-month follow-up, *N* = 68 reduction; 63 maintenance At 7 year follow-up, *N* = 52 reduction; 51 maintenance	Flexible reduction and discontinuation	At 18 months there was no difference in social functioning and an increased risk of relapse among those randomised to reduction	At 7 years, people allocated to reduction showed better social functioning and there was no difference in risk of relapse
Huhn et al., 2020	Schizophrenia or schizoaffective disorder	*N* = 11 reduction; 9 maintenance Follow-up 6 months	Gradual reduction of antipsychotics for 3 months. Average reduction was 42% of baseline dose	No difference in relapse, symptoms, social functioning or side effects at 6 months	N/A
Sturup, 2022	First episode schizophrenia or delusional disorder	*N* = 14 reduction; 15 maintenance Follow-up 1 year	Flexible reduction and discontinuation. Mean 83.9% reduction of baseline dose in tapering group	5 participants in the reduction group and 2 in the maintenance group stopped antipsychotics and remained in remission for at least 3 months. No difference in risk of relapse or social functioning	N/A
Liu, 2023 (GARMED study)	Schizophrenia spectrum disorder	*N* = 51 reduction; 24 maintenance Follow-up 2 years	Hyperbolic reduction. Mean reduction 41% from baseline. No aim to discontinue treatment completely	Reduction group showed greater clinical improvement and improvement in quality of life compared to maintenance group. No difference in relapse risk	N/A
Moncrieff, 2023 (RADAR study)	Recurrent, non-affective psychotic disorders	*N* = 126 reduction; 127 maintenance Follow-up 2 years	Flexible reduction and discontinuation. Median maximal dose reduction 67% in the reduction group	Shorter time to relapse and higher risk of relapse in the reduction group vs maintenance group. No difference in social functioning, symptoms or side effects.	N/A

There have been three studies published so far of significant size. The MESIFOS study in the Netherlands involved a gradual, open, flexible reduction of antipsychotics in people with a first episode of psychosis. After 18 months people randomised to dose reduction were more likely to relapse and showed no benefits in terms of overall social functioning. However, at a seven-year follow-up of 103 participants, people who were randomised to dose reduction were twice as likely to be classified as having made a social recovery than those randomised to maintenance treatment and relapses rates equalised^38^. There was considerable variation in treatment received in both arms over the course of follow-up, but rates of complete discontinuation and use of very low dose antipsychotics were higher at follow-up among those who were originally randomised to dose reduction.

Another trial of gradual dose reduction (25% dose reductions every 6 months, calculated on the most recent dose) was conducted in Taiwan with 96 stable patients with schizophrenia spectrum diagnoses (the GARMED study). It found that people allocated to dose reduction showed improvements in overall clinical improvement scores and quality of life which were not present in the maintenance group, with low rates of relapse which did not differ between groups^11^. This was a dose reduction study with no plan for people to discontinue their medication within the 2-year period of the study^11^.

The RADAR trial (Research into Antipsychotic Discontinuation and Reduction) of 253 people with recurrent psychotic disorders (including schizophrenia) found an increased rate of relapse in those allocated to dose reduction and discontinuation at the two-year follow-up, similarly to the Dutch trial. There were no differences in the primary outcome of social functioning at 24 months, or in any secondary outcomes, including symptom scores, quality of life and side effects^55^. Antipsychotic dose was reduced gradually over 1–2 years with the aim to stop medication when the process proceeded smoothly. 27% of the dose reduction group stopped their antipsychotic medication completely, and 70% reduced the dose by 50% compared with 10% and 17% respectively in the maintenance group. Although numbers stopping antipsychotics completely was small, only 13/47 (27.7%) of them had a severe relapse requiring hospitalisation, and only 32 of the 109 (29.4%) who reduced their dose by at least 50%.

Therefore, evidence on dose reduction and discontinuation using gradual and flexible strategies is mixed. Large studies that involved discontinuation showed increased rates of relapse early on, despite the more gradual nature of reduction than previous studies^38,55^. However, in the one study with long-term follow-up, the risk equalled out with the maintenance group over time^38^ and even when the risk was increased, the majority of people who discontinued or reduced medication did not relapse^55^. Moreover, studies with a lesser degree and slower rate of dose reduction did not show an increase in relapse risk^11,99^. Two studies suggested the benefits of dose reduction at medium or long term follow-up^11,38^.

Some of these studies suggest that patients maintained on lower doses have similar outcomes to patients on higher doses, with less side effects, such as the GARMED study. Several other dose reduction studies have found that use of lower doses was associated with better outcomes than use of higher doses^56,100^. This may be because the pattern of response flattens out at higher doses (due to the receptor occupancy relationship) whereas adverse effects may accumulate. However, some studies find increased rates of relapse or rehospitalisation among people treated with lower compared to higher doses^101,102^ (although these studies involve rapid reductions to lower doses)^103^.

Overall, therefore, the evidence base reveals that not all patients relapse after discontinuing long-term antipsychotic treatment and the benefits may have been overstated due to the adverse effects of withdrawal process itself. The case that antipsychotics target an active or progressive pathological process is unconvincing, the adverse effects of antipsychotics are harmful and burdensome and many patients wish to stop them. These considerations suggest there is a good case for helping people to reduce and discontinue antipsychotics in some situations. Recent studies suggest that a moderately gradual reduction may increase the risk of a subsequent relapse in the short-term, although only a minority relapse even so.

## When and in whom to discontinue antipsychotics

When to stop antipsychotics is another area of debate and again depends on how the evidence on antipsychotics is interpreted. Some current guidelines recommend that people who have had a single episode of psychosis could be considered for medication discontinuation after 1^3^ or 2^5^ years of treatment or remission of symptoms. People who have had multiple episodes of psychosis are advised to stay on antipsychotics for at least 2–5 years^3^, or implicitly indefinitely in many guidelines^4^.

Recommendations about the duration of treatment are based on the duration of maintenance treatment trials, which show that the risk of relapse remains elevated following discontinuation compared to maintenance treatment during trials that last for up to one or two years. The number of trials lasting this long is small, however, with only 6 of the 65 trials in Leucht et al.’s 2012 meta-analysis having longer than a year of follow-up, for example, and then mostly only for a minority of participants^6^.

However, if these are interpreted as showing that long-term treatment induces brain adaptations that increase the risk of relapse after discontinuation through withdrawal-related effects, this would imply that treatment should be tapered and stopped as soon as it can be. For people experiencing a first episode of psychosis or for others who were not taking long-term treatment prior to their episode, this would be as soon as acute symptoms have receded and stability is achieved. Continuing medication for one or two years after remission, as in current recommendations for people with a first episode of psychosis, may exacerbate the risks of withdrawal when the drugs are eventually discontinued. However, social factors are also important considerations, and reducing medication is most likely to succeed when people have adequate social support and stability of housing and income^104^.

Identifying who might be a good candidate for antipsychotic reduction is a long-standing concern. Numerous analyses that have attempted to define which patients are more likely to relapse when antipsychotics are discontinued, but systematic reviews of this research have not produced consistent findings based on demographic or clinical characteristics^105–109^. Certain characteristics of patients may make a process of reduction or discontinuation more feasible in practice, such as good insight into their condition and well characterised early markers of deterioration, with some research finding limited support for the role of early markers^110^. However, the general absence of clear predictors suggests cautious dose reduction could be appropriate in most patients who demonstrate symptom resolution or remission of a first episode, or who demonstrate recovery and stability in multi-episode conditions for a period such as twelve months or more^111^, who do not pose significant risks for violence or suicide, and whose social situations permit. There are also ethical reasons to support such an approach, following the principles of autonomy, as well as beneficence and non-maleficence given the uncertainties outlined above^112^.

## How to discontinue antipsychotics

There has been limited interest in investigating the best way to stop antipsychotics when appropriate on the assumption that the method of stopping is not critical. However, as outlined above, there is some evidence that more gradual cessation of antipsychotics may reduce the relapse rate compared to more rapid withdrawal^41,46^. This may be because gradual cessation allows neuro-adaptations accumulated in response to the presence of antipsychotics to resolve while medication is slowly reduced^12^. Gradual reduction is likely to minimise the difference between what the brain ‘expects’ and the input of the drug. An analogy might be made to altitude sickness, where symptoms arise from too rapid an ascent, leading to an inability of the body to accommodate reduced air pressure. A slower ascent allows the body’s homeostatic capacities enough time to re-adapt slowly to pressure changes^113^. It is the not the extent of the ascent that determines symptoms but that rate of the ascent. It has been suggested by observing the time taken for tardive dyskinesia (the most obvious manifestation of dopaminergic hypersensitivity/neurological adaptation) to resolve after long term antipsychotic exposure, as well as clinical studies and patient experience^114^, that dose reduction may require months to years to allow neuro-adaptations adequate resolution during tapering^12^. Shorter-term users should be able to stop their medications more quickly as adaptations are less well established.

In addition to the rate of dose reduction, the pattern of dose reduction has also been highlighted. It has been long regarded that there is a threshold for antipsychotic action: that blockade of 65–80% of dopaminergic D2 receptors is required for effective antipsychotic action. However, more recently evidence has been presented of a continuous relationship between dose and likelihood of relapse which follows a hyperbolic pattern^115^. A recent dose-dependent meta-analysis found that there is little additional benefit regarding relapse prevention for doses greater than 5 mg risperidone equivalents, and a steeply, hyperbolically increasing risk of relapse as doses reduce below 2.5 mg of risperidone equivalent^116^. This pattern does not conform to the binary threshold model (which would suggest that all or most relapses occur just below the threshold) and is consistent with the hyperbolic pattern of receptor occupancy of antipsychotics, which has also been shown to correlate with symptom reduction^117^. This hyperbolic pattern arises as a consequence of the law of mass action—where small doses of drug have out-sized effects, while increasing doses of drug have incrementally less effect because of receptor saturation^118^. This has led to the idea that hyperbolic dose reductions—in which doses are decreased in incrementally smaller steps to produce equal reductions in receptor occupancy at each step down—are prudent and likely to reduce adverse effects of withdrawal^119^. Indeed, appreciating the hyperbolic receptor occupancy relationship makes it difficult to advocate for linear dose reductions (e.g. 2.5 mg dose reductions per month).

There is emerging empirical support for such an approach, for example the GARMED study, in which doses were reduced in an approximately hyperbolic manner, allowed many patients to reduce their dose by 25–56% without an excess in relapses compared to maintenance groups^11^. Hyperbolic tapering for other psychiatric drug classes has also been demonstrated as more effective than traditional approaches in cohort studies^120^ and recommended in authoritative guidelines such as those by NICE^121^, the Royal College of Psychiatrists^122^ and Australian guidelines^123^. Further details on tapering in this manner are outlined in other papers^12,124^ – but a brief rule of thumb is that many long-term users of antipsychotics may be able to tolerate reductions of 5–10% of their most recent dose per month, where the reductions become smaller and smaller as the total dose gets lower, down to very small final doses, often requiring doses as small as 1% of the smallest commercially available tablets (so that the reduction from the final dose to zero does not cause larger changes in effect on target receptors than previous reductions).

Notably, this specific method of dose reduction (5-10% per month) has not been tested empirically but it is consistent with the approach taken in the GARMED study (25% dose reductions every 6 months)^11^, with the theoretical benefit of smaller steps causing smaller perturbation to the existing homeostatic equilibrium. It is also the approach most favoured to ceasing antipsychotics by patients outside of a medical context, in which patients make very small percentage reductions of their medication over several years to avoid relapse and withdraw successfully^125^. This approach is being evaluated by the DreamsPhen study in France^126^.

Such an approach often involves using formulations of drugs other than widely available tablets and capsules, such as liquids. Skipping doses for drugs which have half-lives of 24 h or less causes problematic variation in plasma levels which can induce withdrawal effects and is not generally recommended. The rate of reduction should be adjusted to the individual—so that signs of withdrawal, such as insomnia, should indicate that the rate of taper should be slowed or paused for a period. However, even if withdrawal symptoms are not readily apparent, by allowing neurological adaptations time to normalize, a hyperbolic approach may reduce the risk of withdrawal-induced relapse at a later date in people who have been on medication for long periods.

There are also numerous psychological aspects of dose reduction. Patients are advised to reduce potential sources of stress and maximise their social support. Patients who reduce their dose may struggle with re-emerging emotions that have been suppressed by medication and potentially complex feelings about past treatment and experiences in mental health services^127^. Gradual, hyperbolic tapering may minimise some of these effects but are unlikely to eliminate them completely and this is an understudied and vital area for future research.

Engagement of patients in a gradual process of reduction and discontinuation might also minimise the well-recognised risks of patients ceasing their antipsychotics abruptly on their own initiative—an approach that is likely to cause the greatest risk of withdrawal effects and relapse—as well as promote a better doctor-patient relationship and potentially curtailing the cycle of cessation, relapse and re-instatement of medication.

## Decision making

What decisions people come to regarding continuing or stopping antipsychotics, and how they should be guided in these, depends on how antipsychotics are deemed to work as well as how evidence for their effects is interpreted.

The logic of the disease-centred model of drug action, and the idea that relapse is a neurotoxic process, is that antipsychotic treatment is necessary to counteract the underlying pathology^67^. Doctors should, therefore, try to persuade their patients to take it even when they don’t want to. Similarly, forcible treatment is easily justified by the idea that it consists of treatment for a disease, with less need to balance the public interest and safety against patients’ wishes.

However, if antipsychotics are understood to work according to a drug-centred model – that is by modifying normal neurological and mental functioning - then every individual will have a different view on whether the benefits of symptom suppression are worth the costs of adverse effects and health complications. The possibility that antipsychotic discontinuation may, in itself, induce a relapse highlights the harmful effects of long-term drug treatment but also the difficulties of stopping it once it is established. Some people will want to avoid symptoms and relapse at all costs, but others may prefer to accept an increased risk of relapse to avoid the negative effects of continuing the drugs and possibly derive benefits in terms of improved functioning and quality of life. Although psychiatrists may generally regard any increase in risk of relapse as being unacceptable^128^ patients may have different perspectives on the relative value of relapse versus long-term functioning and there is evidence that a non-negligible proportion of people can do well on low or no antipsychotics. Additionally, it may be that even more gradual tapering than has been previously employed may lower the risk or relapse even further. The doctor or prescriber’s role is to support the individual in their decision-making to facilitate the individual’s autonomy, maximise beneficence and promote fair and equal treatment (justice)^112^.

This is not necessarily straight-forward, as people with psychosis and schizophrenia sometimes lack capacity to make rational and informed decisions about treatment when acutely unwell, in which case a degree of paternalism may be necessary to judge what is in the individual’s particular interests. Moreover, people with mental disorders can be subject to legal measures which can enforce hospitalisation and medication against the wishes of the individual under certain circumstances, such as when they are felt to be representing a danger to themselves or to other people. Considerations about antipsychotic treatment need to be guided by the need to maintain the safety of others, but there is still an obligation to balance this with the interests of the patient, to consider least restrictive options and to enhance autonomy and facilitate people’s choices within legal limits^112^.

## Conclusions

Accepting that there are different ways to understand antipsychotic action and that long-term treatment may itself contribute to poor outcomes following withdrawal highlights the complexity of the question about whether, when and how to stop antipsychotic treatment. People need support to navigate this complexity in order to make a properly informed analysis of the costs and benefits of ongoing treatment in their individual situation, and doctors need the necessary knowledge and skills to help people do this. We suggest that doctors should be prepared to support people who wish to try to reduce or discontinue their medication, as long as they are able to make a capacitous decision and do not present a significant risk to public or personal safety.

Evidence that long-term treatment may provoke relapse following withdrawal of medication suggests a need to reconsider blanket recommendations for people to remain on treatment for one or two years after the resolution of a first episode. Supporting people to try and gradually stop medication when acute symptoms have subsided and people have achieved a stable condition will reduce the numbers who become established on long-term treatment who do not need it, and minimise future risks of withdrawal effects and withdrawal-induced relapse.

For those who are already established on long-term medication, reducing to lower doses may provide the most favourable balance of benefit and harm. It may be that ‘gold standard’ practice should more assertively involve identifying the minimal effective dose of medication, which balances benefits and harms. This is likely to be lower than previously recognised, and for some patients it may be zero^2^.

If reduction or discontinuation of antipsychotic mediation is undertaken, it should be done slowly and flexibly, following hyperbolic principles at rates as slow as 5-10% per month of the most recent dose for longer term users (with shorter term users able to proceed at faster rates). Monitoring and support are also required so that signs of relapse can be identified and treated, and to help people with other issues that are ignited by withdrawal, such as the return of previously supressed emotions^127^.

## Data Availability

All data associated with this manuscript have been published in the paper.
